# Infection by *Angiostrongylus cantonensis* in both humans and the snail *Achatina (Lissachatina) fulica* in the city of Macapá, in the Amazon Region of Brazil

**DOI:** 10.1590/0074-02760200115

**Published:** 2020-07-06

**Authors:** Tatiane Alves Barbosa, Silvana Carvalho Thiengo, Monica Ammon Fernandez, Carlos Graeff-Teixeira, Alessandra Loureiro Morassutti, Fábio Rodrigo Paixão Mourão, Clóvis Omar Sá Miranda, Michel de Moraes Jorge, Liliane Freitas Costa, Suzete Rodrigues Gomes

**Affiliations:** 1 Secretaria Municipal de Saúde de Macapá, Coordenação de Vigilância em Saúde, Macapá, AP, Brasil; 2 Fundação Oswaldo Cruz-Fiocruz, Instituto Oswaldo Cruz, Laboratório de Referência Nacional para Esquistossomose-Malacologia, Rio de Janeiro, RJ, Brasil; 3 Universidade Federal do Espírito Santo, Centro de Ciências da Saúde, Núcleo de Doenças Infecciosas, Vitória, ES, Brasil; 4 Instituto de Patologia de Passo Fundo, Passo Fundo, RS, Brasil; 5 Superintendência de Vigilância em Saúde, Laboratório Central de Saúde Pública, Amapá, Brasil

**Keywords:** cerebral angiostrongyliasis, eosinophilic meningitis, giant African snail, snail borne diseases, Aelurostrongylus abstrusus, surveillance

## Abstract

In January and February 2019, a malacological survey was conducted in the area surrounding the residence of a 12-year-old child that had contracted cerebral angiostrongyliasis in the municipality of Macapá, capital of the Amapá State, northern Brazil. The serological examination was positive for *Angiostrongylus cantonensis* infection, the principal etiological agent of this parasitosis. A sample of 54 molluscs was artificially and individually digested for parasitological analysis, containing 38 specimens of *Achatina fulica*, nine specimens of *Bulimulus tenuissimus* and seven specimens of *Sarasinula linguaeformis*. *A. fulica* was the most abundant mollusc, and the only species infected with *A. cantonensis*, as well as presenting co-infections with other nematodes. This is the first report of cerebral angiostrongyliasis in the Amazon Region, and the first record of *A. fulica* infected with *A. cantonensis* in Amapá. These findings highlight the potential risks of human angiostrongyliasis, and the need to implement public health measures to control the spread of the disease.

Angiostrongyliasis includes parasitic infections caused by metastrongylid nematodes of the genus *Angiostrongylus* Kamensky, 1905. The life cycle of this nematode includes rodents and other small wild mammals as the definitive hosts and numerous species of terrestrial and freshwater gastropods, which act as intermediate hosts.^1^ ^,^ ^2^ ^,^ ^3^ ^,^ ^4^

Two zoonotic *Angiostrongylus* species are known to occur in Brazil. One, *Angiostrongylus costaricensis* Morera & Céspedes, 1971, causes abdominal angiostrongyliasis, while the other, *A. cantonensis* (Chen, 1935) causes cerebral angiostrongyliasis. The first is found in mesenteric arteries of the caecum of its definitive host while *A. cantonensis*, known as rat lungworm, is found in pulmonary arteries of the host rodents. In these organs the nematodes eggs are laid and hatch releasing the first stage larvae (L_1_) that are released into the faeces of these animals.^5^ ^,^ ^6^ The L_1_ larvae are ingested by molluscs, in which they develop in two weeks into either the second (L_2_) and third (L_3_) stages, depending on the environmental conditions. Rodents become infected through the ingestion of molluscs or plants contaminated with larvae (L_3_) released in the mucus of the mollusc.^6^ ^,^ ^7^ ^,^ ^8^ Paratenic hosts, such as lizards and crustaceans, may also contribute to the continuity of the life cycle of *A. cantonensis*.^9^ Humans may be infected through the intentional or accidental ingestion of contaminated raw molluscs or the paratenic hosts, or inadequately washed fruit or vegetables with mollusc mucus containing L_3_ larvae.^7^ ^,^ ^8^ ^,^ ^9^ ^,^ ^10^ The parasite does not complete its development in humans, however.^4^ ^,^ ^11^

Cerebral angiostrongyliasis, which is also known as eosinophilic meningitis (EoM), is endemic to certain Asian countries and Pacific Islands.^12^ In recent years, distinct species of intermediate and definitive hosts have been found to be infected with *A. cantonensis* outside its original area of occurrence, including countries in the Caribbean region and in South America, such as Brazil.^4^ ^,^ ^6^ ^,^ ^12^ ^,^ ^13^ ^,^ ^14^ In Brazil, cerebral angiostrongyliasis was first reported from Cariacica and Vila Velha, in the State of Espírito Santo,^15^ and Recife, capital of the State of Pernambuco,^1^ ^,^ ^2^ although there are now many recorded cases in humans from other regions of the country, including the states of São Paulo^16^ and Rio Grande do Sul.^7^ Given this context, cerebral angiostrongyliasis is now considered an emerging disease in Brazil, with 35 confirmed and 84 suspected cases.^17^ The worldwide dispersal of rodents contributes to the spread of *A. cantonensis*,^16^ as does the expansion of the distribution of exotic molluscs, such as *Achatina* (*Lissachatina*) *fulica* Bowdich, 1822, known as the giant African snail.^18^ The lack of natural enemies and pathogens for this exotic species, combined with the ongoing effects of climate change, contribute to the adaptability of *A. fulica* to novel environments, which had led to its proliferation in many areas of Brazil.^9^ ^,^ ^14^ ^,^ ^18^

The present study provides the first report of a case of cerebral angiostrongyliasis in the Brazilian Amazon Region, in the municipality of Macapá, capital of the Amapá State. The study also presents the first record of *A. cantonensis* larvae infecting the giant African snail, *A. fulica*, in Amapá.

The epidemiological investigation in Macapá was initiated in August 2018, one month after a 12-year-old child had tested positive for cerebral angiostrongyliasis. The diagnosis was based on a combined ELISA and Western blot to identify the diagnostic antigen of the 31kDa band.^7^

Two months after the diagnosis, the area around the patient’s house in the Santa Rita neighborhood of Macapá was surveyed, although no molluscs were found. The patient’s mother then reported that the child had had contact with African snails on a vacant lot near the family home, which led to the expansion of the search to five additional sites within an area of approximately 1 km^2^ around the patient’s house, in January and February 2019. The African giant snail was the focus of the epidemiological study, based on the mother’s patient report. A sample of 54 molluscs was artificially and individually digested for parasitological analysis, containing 38 specimens of *A. fulica*, nine specimens of *Bulimulus tenuissimus* (d’Orbigny 1835) and seven specimens of the slug *Sarasinula linguaeformis* (Semper, 1885). Samples of these species were also fixed and deposited in the mollusc collection of the Oswaldo Cruz Institute (CMIOC) for anatomical analysis and taxonomic identification: *A. fulica* (CMIOC 11799, three specimens; CMIOC 11804, one specimen), *B. tenuissimus* (CMIOC 11816, one shell), and *S. linguaeformis* (CMIOC 12388, two specimens). As only few specimens of the *Subulina octona* (Bruguière, 1789) (CMIOC 11803, six specimens), *Allopeas gracile* (Hutton, 1834) (CMIOC 12315, five specimens) (both micromolluscs species), *Succinea* sp. (CMIOC 11802, two juvenile specimens) and *Solaropsis rosarium* (Pfeiffer, 1849) (CMIOC 11800, one specimen; CMIOC 11801, four specimens) were found, we used these samples just for taxonomic identifications based on morphology.

The parasitological examinations were based on the following protocol (modified from Wallace & Rosen 1969):^19^ the soft tissue was triturated and digested in a 0.7% solution of HCl for 6 h, with the resulting liquid being filtered through a Baermann funnel for the isolation of the third stage larvae (L_3_), which were examined under a stereomicroscope. All the larvae with morphological traits of the superfamily Metastrongyloidea were separated for analysis, and those with characteristics of *Angiostrongylus* spp. were analysed using molecular techniques for species identification. The DNA was extracted by the thermal shock method, with up to 10 larvae, in microcentrifuge tubes, being immersed in liquid nitrogen for 1 min and then being transferred to a dry bath at 95ºC for 20 min. This process was repeated three times for each sample.

The polymerase chain reaction (PCR) had a volume of 50 μL, containing 23.90 μL of ultrapure water, 11 μL of 10% trehalose, 5.5 μL of 10x PCR reaction buffer, 4.4 μL of 2.5 mM dNTPs, 2.75 μL of 50 mM MgCl_2_, 1.1 μL each of 5 μM the forward and reverse primers (Nem 3) from Prosser et al.,^20^ and 0.25 μL of recombinant Taq DNA polymerase (Thermo Fisher Scientific). A 5 μL aliquot of the DNA was added to the mixture to reach a final volume of 55 μL for each reaction. The PCR products were purified using the Illustra GFX PCR DNA and Gel Band Purification kits (GE Healthcare, Little Chalfont, UK) following the manufacturer’s protocol. The purified products were sequenced bidirectionally, using the BigDye Terminator v3.1 Cycle Sequencing kit (Applied Biosystems, California, USA) according to the manufacturer’s instructions, on the RPT01A Genomic Platform at the Oswaldo Cruz Institute, in Rio de Janeiro. The sequences obtained in the present study were deposited in GenBank. The chromatograms of the sequences were analysed and edited using Geneious version R9 (http://www.geneious.com), which resulted in a consensus sequence (contig), which was compared with reference sequences from GenBank using the BLASTn algorithm.^21^

The three analysed species in the parasitological investigation, *A. fulica*, *B. tenuissimus* and *S. linguaeformis*, are synanthropic, widely distributed in Brazil and known as natural hosts of *A. cantonensis*.^5^ ^,^ ^9^ ^,^ ^22^ ^,^ ^23^ The invasive giant African snail *A. fulica* has also being showed to have an important role as intermediate host of this nematode in urban areas because of its extensive presence in many parts of Brazil and its susceptibility to *A. cantonensis* infection.^1^ ^,^ ^3^ ^,^ ^4^ ^,^ ^7^ ^,^ ^9^ Oliveira et al.^24^ examined seven terrestrial mollusc species in the urban zone of the city of São Gonçalo (State of Rio de Janeiro), and two of these species ― *A. fulica* and *Bradybaena similaris* (Férussac, 1821) ― were parasitised by *A. cantonensis*. In this study, the prevalence of *A. cantonensis* in *A. fulica* was more than 50% while in *B. similaris*, it was 24.6%, and both species were also co-infected with other helminths. In the metropolitan region of Aracaju (Aracaju, Barra dos Coqueiros, Nossa Senhora do Socorro, and São Cristovão), *A. cantonensis* infection rates were 17.7% in *A. fulica* and 4.3% in *B. tenuissimus*,^25^ which further reinforces the parasitological importance of these molluscs. Specimens of the slug *S. linguaeformis* have also been found to be infected with both *A. cantonensis* ^5^ and *A. costaricensis*. The parasitological examination of 50 *S. linguaeformis* specimens collected in rural Nova Itaberada, a municipality in the southern Brazilian State of Santa Catarina, also revealed infection by *A. costaricensis* in 43 of the 50 slugs.^26^

Of the three species analysed by artificial digestion in the present study, only *A. fulica* was found to be infected with parasites of the superfamily Metastrongyloidea, with 36 of the 38 specimens analysed being infected. Two species of parasite were identified. One was *A. cantonensis*, identified based on the COI sequences obtained here (571-611 base pairs), which were deposited in GenBank (MN994436, MN994437 and MN994438). These sequences were 100% similar to those of *A. cantonensis* available in GenBank (MH511542.1 from Brazil, MH511542.1 from Austrália, MK570629.1 from Spain, MF000735.1 from United States, and AP017672.1 from Japan). The second parasite species, *Aelurostrongylus abstrusus* (Railliet, 1898), was identified based on its morphology. Molluscs infected with *A. cantonensis* and *A. abstrusus* were collected at three and two of the five study sites, respectively.

This confirms the participation of *A. fulica* in the life cycle of these nematodes, as already observed in other Brazilian states.^5^ ^,^ ^6^ ^,^ ^25^ *A. fulica* has been reported previously in the city of Macapá, in the northern (Brasil Novo neighborhood), central (Central neighborhood), and southern (Pedrinhas neighborhood) zones,^27^ although this study did not include parasitological analysis. In northern Brazil, *A. fulica* and rodents have been found infected with *A. cantonensis* in the Guamá and Jurunas neighborhoods of the city of Belém,^13^ although no human infections have been reported.

The discovery of *A. abstrusus* parasitising *A. fulica* in Macapá serves as an alert for veterinary medicine, given that this nematode species is a lungworm of cats with a worldwide distribution,^28^ including Brazil.^23^ ^,^ ^29^ ^,^ ^30^ The parasite infection rate recorded in *A. fulica* in the present study (94.7%, that is, 36 of the 38 snails examined were infected) was higher than those recorded in previous studies,^24^ which highlights the potential role of this exotic species in the transmission of *A. cantonensis* and *A. abstrusus* both of veterinary and public health importance. It is also interesting to note that four *A. fulica* specimens had a double infection, with *A. cantonensis* being associated with either *A. abstrusus* or a third, yet unidentified nematode, which further reinforce the urgent need for the control of this exotic species in Brazil.

This is the first report of *A. fulica* being infected with *A. cantonensis* in the Brazilian State of Amapá and the first record of a case of cerebral angiostrongyliasis in the Amazon Region. These findings stress the importance of raising the awareness of public health services with regard to the presence of cerebral angiostrongyliasis in northern Brazil. Preventive measures include the avoidance of contact with potentially infective molluscs, which can be encouraged through health education initiatives. Clearly, further research is urgently needed to map the potential distribution of *A. cantonensis* in Macapá and in other areas in the Amazon Region.
